# Acute Oculomotor Nerve Palsy Caused by Compression from an Aberrant Posterior Communicating Artery

**DOI:** 10.7759/cureus.3920

**Published:** 2019-01-19

**Authors:** Ali S Haider, Caleb Gottlich, Hasan Sumdani, Kennith F Layton, Kyle Doughty

**Affiliations:** 1 Neurosurgery, Texas A&M College of Medicine, Houston, USA; 2 Orthopaedics, Texas A&M College of Medicine, Dallas, USA; 3 Neurosurgery, Texas A&M College of Medicine, Round Rock, USA; 4 Radiology, Baylor University Medical Center, Dallas, USA; 5 Neurosurgery, Baylor University Medical Center, Dallas, USA

**Keywords:** aneurysm, palsy, posterior communicating artery, oculomotor nerve, neurosurgery, vascular, cranial nerve, nerve compression, aneurysm clip, anatomy

## Abstract

Oculomotor nerve palsy (ONP) is a rare neurological deficit associated with numerous underlying pathologies. Of these, aneurysm of the posterior communicating artery (PCOM) has been described due to the intimate association with the third cranial nerve in the basal cistern. Less described are other vascular abnormalities and their contribution to ONP. We describe a case of ONP thought to result from a PCOM aneurysm, per the associated magnetic resonance imaging (MRI) scan, yet found intraoperatively to be caused by a congenital vascular aberrancy. This is the first case, to our knowledge, of this presentation.

## Introduction

Isolated cranial nerve palsy frequently involves the third cranial nerve due to its anatomic surroundings when leaving the brainstem in addition to its precarious positioning between the uncus and the tentorium. When presenting acutely, palsy of the third cranial nerve is often a harbinger of an unstable aneurysm. These symptomatic aneurysms frequently arise from the posterior communicating artery (PCOM), which is the most common type of aneurysm to form in the basal cistern. The majority of symptomatic aneurysms of the PCOM present as an oculomotor nerve palsy (ONP), which can develop directly via mass effect of the growing aneurysm or indirectly via rupture of the aneurysm [1]. The acute presentation of these cases is indicative of aneurysmal instability and often heralds impending rupture, thus denoting a need for immediate investigation. Although only a minority of ONP is caused by aneurysm, it is still imperative to rule out this diagnosis [2]. Several features of the history and physical exam can be used as supporting evidence for or against aneurysmal ONP and should be noted prior to a full work up. Imaging such as magnetic resonance angiography (MRA), computed tomography angiography (CTA), and digital subtraction angiography (DSA) are considered the gold standard for evaluating potential ONP due to aneurysm. However, these imaging modalities can still be fallible in certain instances. Here, we offer an example of a woman presenting with ONP suspected to be attributable to aneurysm of the PCOM but intraoperatively was found to be caused by a tortuous PCOM. To our knowledge, this is the only report of such a presentation.

## Case presentation

**Figure 1:**
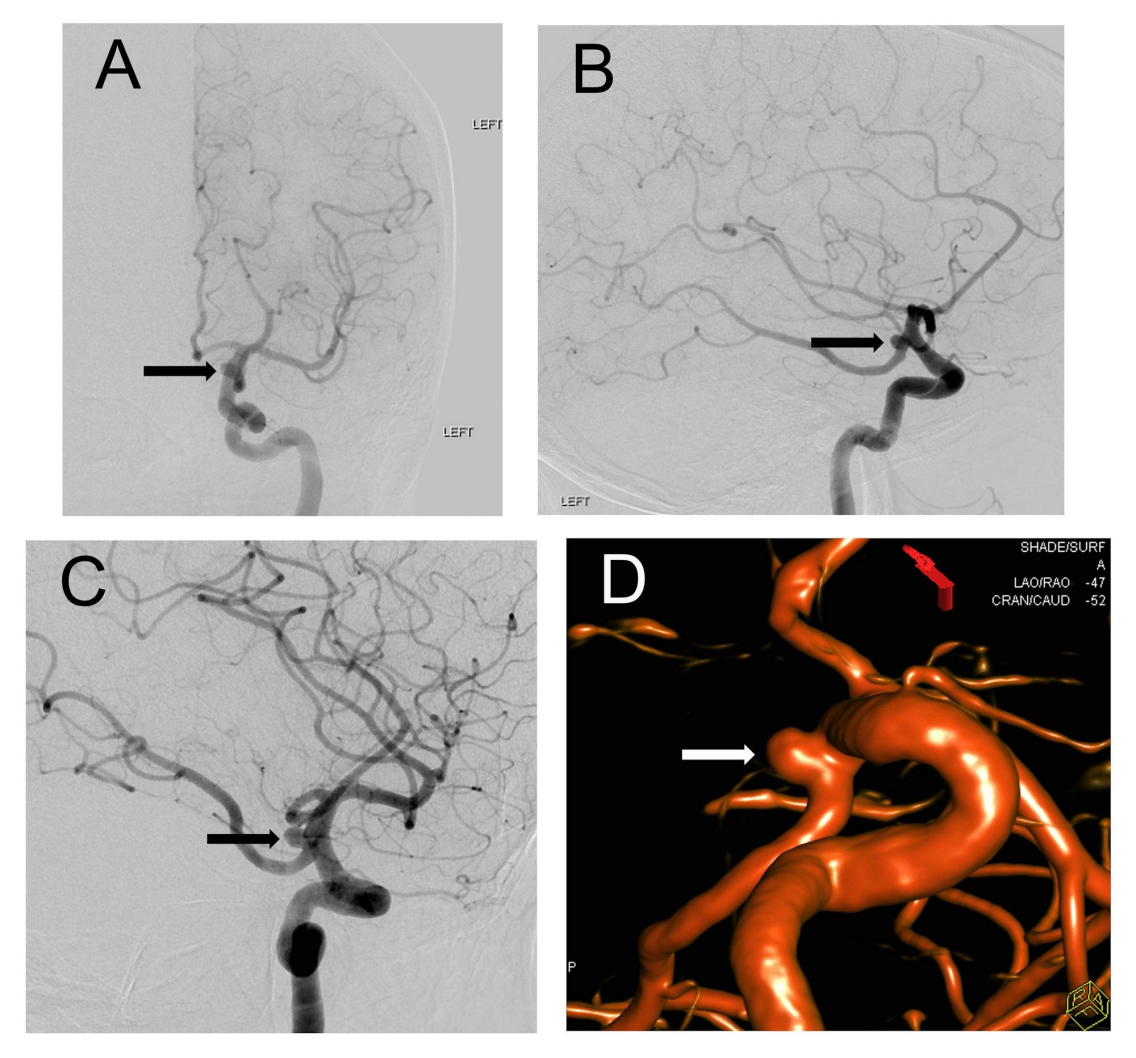
Digital Subtraction Angiography (DSA) with 3-Dimensional Reconstruction Frontal and lateral view (A and B) of left internal carotid artery digital-subtraction angiogram (DSA) demonstrating the medially projecting aneurysm (arrows) arising from the origin of a fetal type posterior communicating artery. Oblique working projection DSA (C) image from left internal carotid artery injection again demonstrates the left posterior communicating artery aneurysm (arrow). A 3-dimensional computer reconstruction of the magnetic resonance angiogram images further denotes the aneurysm (D) (arrow).

A 76-year-old Caucasian woman with a past medical history of hypertension, hypercholesterolemia, and tobacco use presented with a three-week history of progressively worsening left-sided incomplete ptosis. This was accompanied by discomfort in the occipital and upper cervical region. She denied vision changes and eye pain. Her pupils were equal, round, and reactive to light and accommodation, and she displayed no other focal neurological deficits on physical exam. Brain magnetic resonance imaging (MRI) and MRA performed prior to the acute presentation of symptoms showed a 4 mm ipsilateral left PCOM aneurysm. Cerebral DSA performed on admission confirmed a fetal left PCOM with a broad-necked, smooth-walled 4 mm unruptured aneurysm at its origin (Figure 1).

**Figure 2:**
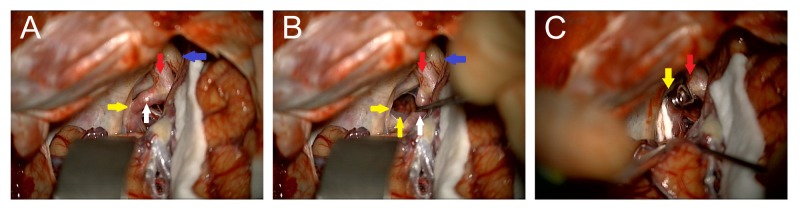
Intraoperative Clipping Intraoperative image showing the optic nerve (blue arrow), proximal internal carotid artery (red arrow), and the fetal posterior communicating artery (PCOM, white arrow) indenting and displacing the cranial nerve (CN) III (yellow arrows) laterally (A and B). The clipped aneurysm and Teflon felt between the PCOM and CN III are shown (C).

Given her recent symptom onset and clinical worsening, the decision was made to perform a left craniotomy with clipping of the aneurysm. Intraoperatively, the non-aneurysmal portion of the left fetal PCOM was found to be compressing the left oculomotor nerve laterally, thus microvascular decompression was performed with a felt pledget, similar to the approach taken in a case of trigeminal neuralgia (Figure 2).

The patient tolerated the surgery well and was discharged to rehab after an uneventful postoperative course. On clinical follow-up, her ONP had completely resolved.

## Discussion

This case is unique in that the patient presented with image verified aneurysm in addition to symptomatology congruent with this finding, but upon intraoperative evaluation, was found to have oculomotor nerve compression more likely attributable to a tortuous PCOM. Upon clipping of the aneurysm and placement of a felt pledget between the aberrant artery and nerve in question, amelioration of symptoms was appreciated. To our knowledge, this procedure has not been documented for use in a similar manner.

This patient presented atypically to most cases of oculomotor nerve compression due to the lack of pupillary involvement, thus suggesting underlying microvascular pathology. If ONP is present with pupillary involvement, there is a higher suspicion of compression because the pupillomotor fibers as well as their vascular component derived from overlying pia course along the superficial, superomedial aspect of the oculomotor nerve [3]. Other common causes of ONP include trauma, neoplasms, stroke, post-surgical inflammation, and microvascular damage from chronic disease, with microvascular damage being the most common [4]. Though these can frequently be evaluated with a thorough history and physical, supporting imaging is often the most reliable diagnostic modality and it should be obtained.

This case also highlights the advantages in certain clinical scenarios to clipping of aneurysms over coiling. In this diagnosis, clipping is typically preferred due to the ability to alleviate aneurysmal mass effect and increased the likelihood of complete or partial ONP resolution, as compared to coiling [5-6]. In addition, this case serves as a demonstration of the potential fallibility of neurovascular imaging techniques, though they are considered the diagnostic gold standard. Although quite rare, aberrant anatomy should be included in a differential in order to offer a potential explanation for inexplicable incongruences in imaging that often requires further evaluation with high-resolution MRI [3]. Radiological evaluation of third cranial nerve palsies remains an exceedingly difficult task and should be done by an experienced, fellowship trained neuroradiologist.

Palsy of the third cranial nerve by anatomic aberrancy is an exceedingly rare condition, with variation of the posterior cerebral artery more commonly documented. To our knowledge, there are eight documented cases of aberrant PCA causing ONP with no examples in the literature of aberrant PCOM causing ONP [7]. This case offers an efficacious approach to the resolution of ONP caused by compression of a tortuous PCOM.

## Conclusions

Early recognition and evaluation of palsy of the third cranial nerve is important in order to rule out a potential PCOM aneurysm. Aberrant vasculature of the PCA or PCOM are rare but are possible causes of ONP and thus should be kept in the differential for these patients, especially when supporting imaging is found to be noncontributory. Here, we discussed a case in which a woman presented with acute onset ONP and vascular imaging corroborated the diagnosis of a PCOM aneurysm. Intraoperatively, the neurosurgeon was confident in attributing the cause to a tortuous PCOM compressing the oculomotor nerve and noting the aneurysm as merely an incidental finding. Microvascular decompression was achieved with a novel approach of placing a felt pledget between the artery and nerve with concurrent clipping of the aforementioned aneurysm. Full return of oculomotor function was obtained with no lasting deficit. To our knowledge, this unique presentation of abnormal vasculature compressing the oculomotor nerve resulting in nerve palsy has not been previously identified.
